# Call to Action for Nurses/Nursing

**DOI:** 10.1155/2016/3127543

**Published:** 2016-04-06

**Authors:** Shahirose S. Premji, Jennifer Hatfield

**Affiliations:** ^1^Faculty of Nursing, University of Calgary, 2500 University Drive NW, Calgary, AB, Canada T2N 1N4; ^2^Department of Community Health Sciences, Cumming School of Medicine, University of Calgary, TRW Building, 3rd Floor, 3280 Hospital Drive NW, Calgary, AB, Canada T2N 4Z6; ^3^O'Brien Institute for Public Health, Calgary, AB, Canada T2N 4Z6; ^4^Alberta Children's Hospital Research Institute, Calgary, AB, Canada T2N 4N1; ^5^Institute for Gender Research, Calgary, AB, Canada T2N 1N4

## Abstract

The 13 million nurses worldwide constitute most of the global healthcare workforce and are uniquely positioned to engage with others to address disparities in healthcare to achieve the goal of better health for all. A new vision for nurses involves active participation and collaboration with international colleagues across research practice and policy domains. Nursing can embrace new concepts and a new approach—“One World, One Health”—to animate nursing engagement in global health, as it is uniquely positioned to participate in novel ways to improve healthcare for the well-being of the global community. This opinion paper takes a historical and reflective approach to inform and inspire nurses to engage in global health practice, research, and policy to achieve the Sustainable Development Goals. It can be argued that a colonial perspective currently informs scholarship pertaining to nursing global health engagement. The notion of unidirectional relationships where those with resources support training of those less fortunate has dominated the framing of nursing involvement in low- and middle-income countries. This paper suggests moving beyond this conceptualization to a more collaborative and equitable approach that positions nurses as cocreators and brokers of knowledge. We propose two concepts, reverse innovation and two-way learning, to guide global partnerships where nurses are active participants.

## 1. Background

The eight Millennium Development Goals (MDGs), established during the Millennium Summit of the United Nations in 2000, marked a pledge by 189 nations to foster international relations with shared values of freedom, equality, solidarity, tolerance, respect for nature, and shared responsibility [1]. As global citizens determined to accelerate progress towards MDGs, nurse clinicians have been sharing knowledge and partnering with colleagues in low- and middle-income countries to identify effective ways of working within the context of their healthcare systems. The practice-based process and strategies applied to improve individuals', organizations', or communities' ability to address health issues is referred to as capacity building [2, 3]. Historically, this process was characterized by knowledge transfer from “north to south” or from “developed to developing countries” or “colonizer to colonized” and was framed as “transporting” of knowledge to build capacity [4, 5] in different parts of the globe; it is now passé. Recently, global health orientations that seek to address these enduring approaches to north-south collaborations have challenged the unidirectional flow of knowledge and skills [6] and follow more equitable partnership models [2]. We urge the nursing community to embrace a new ideology that is not based on or concerned with distinctions—north and south, low- and middle-income countries and high-income countries, and developing and developed countries— but is rather concerned with One World, One Health. This opinion paper explains the concept One World, One Health and takes a historical and reflective approach that invites consideration of concepts that can inform the way nursing responds to the challenges in global health engagement. We examine terms such as capacity building and explicate how they have been reframed and advanced in the global health and nursing literature including the transformative nature of concepts such as reverse innovation and two-way learning. We conclude by examining implications for nursing, and policy to instill a new perspective—One World, One Health.

## 2. One World, One Health

Beyond the expiry of the MDGs, it is argued that “the health of people in all countries” must be an overarching goal of a post-2015 framework [7]. Emerging trends, for instance, majority (70%) of the world's poor now reside in middle-income countries [7], and associated challenges necessitate applying post-2015 goals to all countries, with modification of targets and indicators depending on country context [7]. The sentiment of our shared health concerns and coming together of experts and disciplines to address global challenges is implied in the term “One World, One Health.” One World, One Health was coined in 2008 in reference to a consultation document reviewed at the Sixth International Ministerial Conference on Avian and Pandemic Influenza in Egypt [6]. One World, One Health typifies our interconnectedness, between not only humans but nonhumans (i.e., animals) and our ecosystem and emphasizes coequal collaborations and partnerships [6], providing a privileged intersection in which the capacity of healthcare providers, regardless of geographical boundaries, is enhanced for society's well-being [8].

One World, One Health is a relatively new frame of reference in nursing, despite nurses' unique position to address disparities in healthcare to attain health for all people around the world. There are 13 million nurses worldwide who are front-line healthcare providers and, by virtue of their roles and responsibilities, have prolonged encounters with patients and their families [9]. Nurse clinicians require an inquiry approach situated within a cultural-competency framework that promotes behaviors, attitudes, and practices to come together in interpersonal and interprofessional relationships that (a) acknowledge and view cultural differences as strength, (b) promote self-reflection to develop an understanding of their own culture, attitudes, and prejudices, (c) avoid assumptions and stereotypes, and (d) facilitate empathy, despite language or communication barriers [10, 11]. Moreover, knowledge, skills, attitudes, and core values of nurses are fluid [5]—borderless. We assert that for the nursing profession to occupy a forceful role in promoting the aspirations of the Sustainable Development Goals we must see ourselves as partners with our international colleagues, cocreating knowledge and sharing ideas and best practices with a view to seeking innovative solutions to shared health challenges.

## 3. Transformative Nature of Knowledge Transfer, Reverse Innovation, and Two-Way Learning

The term capacity building, dominant in the development literature since the 1970s, has a historical trajectory informed by colonial perspectives and unequal power relationships. Capacity building has been reframed to include notions such as working collaboratively to enhance people's leadership and commitment (i.e., dynamic capabilities) to effect change in the conditions of their communities through action (e.g., discovering new ways of doing things) or responsiveness to the changing environment [3, 12–14]. Moving beyond the colonial perspective to a more ethical, equitable approach leads to framing this process in terms of the quality of the relationships that are nurtured between nurses. This spirit and approach are best characterized by the concept of knowledge transfer [4]. Knowledge transfer promotes access to new knowledge, generally created through research, to those who will use this knowledge. The use of this knowledge is intended to improve outcomes of health and ensure effective use of resources and time [4, 15]. Knowledge transfer entails a social process in which a knowledge manager or broker seeks out existing evidence or seeks to bring research activities more in line with users of knowledge [4, 15]. Nurses need to participate in new ways within this social process, developing competencies to promote social, economic, and political action that not only exposes the health inequalities (e.g., social determinants of health) [16] but identifies innovative approaches to reform healthcare delivery. The knowledge transfer experience should be transformative to all individuals engaged in the experience: creator of knowledge, broker of knowledge, and user of knowledge.

With growing awareness of the transformative nature of international activities, concepts such as reverse innovation and two-way or shared learning have also been advanced in the literature. Reverse innovation is a term that appeared in 2009 and is more prominent in the business literature. It entails applying successful innovative approaches originating from low- and middle-income countries (driven by limited resources) to high-income countries in order to transform healthcare systems and improve health outcomes of patients and communities [17–19]. For example, North Wales implemented a Brazilian family health strategy—universal primary care services—in which primary care teams comprising a doctor, nurse, nurse auxiliary, and four community health workers delivered primary care services to households within their defined geographic area, whether or not the household express a need for service [20]. A number of barriers, however, influence reverse innovation, including weak flow infrastructure, narrow-mindedness, and early failures [17]. Overcoming these barriers is important to realize that the goal of reverse innovation is “to contribute to the countless health challenges faced by populations across the world” [17, p. 2]. The 13 million nurses worldwide can promote a “global innovation flow” that is bidirectional, sharing knowledge, skills, ideas, and lessons learned around the world in order to cocreate clinical practice solutions for the world [17, 21]. Moreover, this global innovation flow should be linked to the Sustainable Development Goals (e.g., “ensure healthy lives and promote well-being for all at all ages”) in a way that seeks to achieve economic, social, and environmental development with hopes of eliminating all forms of poverty [17, 22]. As such, nurses need to embrace new ways of doing, a paradigm shift, which entails examining problems across situations or contexts, being inclusive of other disciplines to explore the complex nature of the problem, and finding alternate and creative solutions. At the forefront of these solutions should be the principles of dignity, prosperity, justice, partnership, planet, and people [7].

Similarly, two-way learning is synonymous with reverse capacity development, which refers to the altered perception, particularly awareness and understanding, of these perspectives or worldviews that can be “translated” into action that can potentially inform practice “at home” [14]. Only one article in the policing literature precisely referred to the concept of reverse capacity development [14]. The authors reflected on Australian police officers' experiences related to peace keeping and capacity building in Solomon Islands, Timor-Lester, and Papua New Guinea. Harris and Goldsmith [14] refer to a “positive effect” of these reverse capacity building experiences, such as enhanced repertoire of skills to improve structures or processes in their workplace, that go beyond enhancing one's own clinical and cultural-competency skills.

Cross-cultural competencies are one strategy to improve patient outcomes and eliminate racial and ethnic disparities in health outcomes [10]. Health inequities, however, are also rooted in social determinants of health (e.g., social status, income, gender, disability, or sexual orientation) [23]. Nursing involvement in the global innovation flow offers an opportunity to support learning in utilizing the social determinants of health framework “at home” to increase access to not only quality care but health resources thereby reducing health inequities [23, 24]. Indirectly, it increases nursing workforce diversity, which has been identified as an important strategy to overcome health inequities [23, 24]. Nursing engagement in the “global innovation flow” is an innovative strategy to reduce inequality within countries—nursing's contribution to moving towards Sustainable Development Goal (e.g., Goal 10 “reducing inequality within and among countries”) [1]. To ensure an effective healthcare system that is accessible, safe, effective, and affordable around the world, nurses also need to change the conversion to influence policy (health and social) [25].

## 4. Nursing: Engagement in Changing Conversation

Nurses have the potential and often the interest to participate in addressing many of the global health issues (e.g., noncommunicable disease) [9] through engagement in areas of healthcare reform that are common across all countries, despite contextual differences [26, 27]. They are uniquely positioned to facilitate shared learning globally and engage in reverse innovation and reverse capacity development (i.e., two-way learning). Reports and studies [9, 26–28] suggest that nurses are viewed as trusted professionals who have the ability to influence elements of healthcare reform. Though there is an appreciation that nurses will need to increase their visibility in shaping international practice and policy decisions (e.g., international agencies, national capitals) [9, 26–28]. The International Council of Nurses realizes that nurses will need to better understand the global health discourse and shape and reshape the conversations at multiple levels (i.e., intrapersonal, interpersonal, organizational, and sociocultural) to inform world views and promote behavior change, that is, involvement in healthcare reform that will lead to health for all [27]. The Global Advisory Panel for the Future of Nursing convened by Sigma Theta Tau International has created a platform for these conversations to increase nurses' contribution to global health [29].

Reverse innovation and two-way learning create an openness to “change the conversation” [30], that is, engage in discourse to promote change in thinking and behavior (i.e., taking action in global healthcare reform). Reverse innovation and two-way learning can promote respect for intellectual partnerships and shared exchange of knowledge, ideas, skills, and innovation across borders [19]. It does not, however, dispel the power dynamics or the view of them and the “other.” Moreover, reverse innovation and capacity development although helpful concepts are not sufficient and must be complemented by an understanding that embodies the complex interrelationship between community engagement and core values of social responsibility, justice, and equity.

## 5. Implications for Nursing and Health Policy

Access to high-quality and high-value care should be a fundamental right of every patient, regardless of the country in which they are receiving healthcare services. The post-2015 United Nations Sustainable Development Goals aspire to this agenda in ways that differ from the “donor-recipient” paradigm of the MDG as it empowers every individual to action [7, 31]. Participation of nurses, a key principle of the Sustainable Development Goals, at every level (see Table 1) will be imperative to reform healthcare to move towards this agenda (i.e., improving access and quality while making healthcare affordable) [32].

Nursing academic and professional institutions are integral to creating an enabling environment for nurses to develop the skills and competencies to participate in addressing inequities in health and healthcare delivery. Professional nursing education programs must help nurses develop competencies (e.g., political leadership, team work) and attributes (e.g., influence, professional credibility) that are fundamental for nursing engagement in global health and health reform [32–34]. To improve health system performance, leadership, critical reasoning, and data management skills are required to generate and use data to inform decisions regarding clinical, research, and education practice and policy [25, 33]. Nurses are apt at adopting and implementing policy but appear to be peripheral in informing and shaping policy [35]. Interprofessional education to promote networking, collaboration, nonhierarchical relationships, and common goals will resolve issues related to professional silos and exclusion of nurses at the policy table [32, 33]. Emotional intelligence (i.e., self-awareness and social astuteness) will enable nurses to manage social and cultural factors that impede their involvement in promoting changes in practice, education, and policy [32]. Professional institutions maintain the responsibility of (a) ensuring nursing presence during policy decision-making, (b) preserving a united front, and (c) guiding nurses to remain proactive in lobbying government and stakeholder to address social determinants of health, which influence health, and access to healthcare [32]. Nurses will need to hold governments, nongovernmental organizations, private sectors, and academic and professional institutions, among others, accountable to the commitments made in delivering priorities in the Post-2015 Development Agenda [36].

## 6. Conclusion

This paper intends to begin conversation among nurses about their engagement in global health and identify actions to participate in health reform required to promote health for all. Grounding nursing conversation in the concepts articulated above will guide change [30], in that it will enable nurses to be accountable to both patient care and healthcare leadership [27]. In so doing, nurses can influence organizational and political context of care, cocreate global solution for care through “global innovation flow,” and emphasize primary care to promote well-being of populations [27]. Disengagement, on the other hand, will result in preserving the status quo and increase healthcare spending without improvement in patient and population well-being.

## Figures and Tables

**Table 1:** Key recommendations for nursing engagement in global health.

Stakeholder group	Recommendation
Nurses	Need to participate in the conversation at every level (e.g., academic, association, and policy) and develop emotional intelligence
Professional nursing education programs	Help nurses develop competencies and attributes for engagement in global health reform
Interprofessional education programs	Promote networking, collaboration, nonhierarchical relationships, and common goals
Nurse clinicians	Need inquiry approach situated within a cultural-competency framework
Nursing community	Embrace One World, One Health
